# Effect of high-fat feeding on expression of genes controlling availability of dopamine in mouse hypothalamus

**DOI:** 10.1016/j.nut.2009.05.007

**Published:** 2010-04

**Authors:** Alex K. Lee, Marjan Mojtahed-Jaberi, Theodosios Kyriakou, Estibaliz Aldecoa-Otalora Astarloa, Matthew Arno, Nichola J. Marshall, Susan D. Brain, Sandra D. O'Dell

**Affiliations:** a Nutritional Sciences Division, King's College London, London, United Kingdom; b Genomics Centre, King's College London, London, United Kingdom; c Cardiovascular Division, King's College London, London, United Kingdom

**Keywords:** Gene expression, Microarray, Dopamine, Diet-induced obese mouse, Hypothalamus

## Abstract

**Objective:**

Hypothalamic centers integrate external signals of nutrient availability and energy status and initiate responses to maintain homeostasis. Quantifying changes in hypothalamic gene expression in the presence of nutrient excess may identify novel responsive elements.

**Methods:**

Affymetrix Mouse Genome 430 2.0 oligonucleotide microarrays containing 45 102 probe sets were used to interrogate differential expression of genes in dietary-induced obesity model C57BL6 inbred mice fed a high-fat (35% fat; *n* = 8) or standard (4% fat; *n* = 6) diet from 3 to 15 wk of age. Ontologies of regulated genes were examined and expression of selected genes was validated by quantitative real-time polymerase chain reaction.

**Results:**

One thousand two hundred twelve unique gene transcripts showed altered expression on the microarrays. Gene ontology analysis revealed changes in neuropeptide genes responding to leptin, *Pomc*, *Cart*, *Npy*, and *Agrp,* compatible with a homeostatic response to high-fat intake, although mean weight increased 2.3-fold compared with standard fed mice (*P* < 0.001). Neurotransmitter system ontologies revealed upregulation of five genes controlling availability of dopamine. Changes in *Th* tyrosine hydroxylase (2.1-fold) and *Slc18a2* solute carrier family 18 (vesicular monoamine), member 2 (4.4-fold) controlling synthesis and release, and *Slc6a3* solute carrier family 6 (neurotransmitter transporter, dopamine), member 3 (4.8-fold), *Snca* α-synuclein (1.3-fold), and *Maoa* monoamine oxidase (1.9-fold) limiting availability were confirmed by quantitative real-time polymerase chain reaction.

**Conclusion:**

Expression of five genes involved in availability of dopamine was increased after a high-fat diet. Failure to reduce dopamine availability sufficiently, to counter the feeding reward effect, could contribute to diet-induced obesity in these mice.

## Introduction

Energy homeostasis is maintained by multiple mechanisms that gather information on nutritional status and make appropriate behavioral and metabolic responses to changes in fuel availability [1]. Hormones such as leptin and insulin and nutrients such as glucose and fatty acids are key peripheral messengers, which communicate energy status to hypothalamic centers involved in energy homeostasis: the arcuate nucleus (ARC), ventromedial nucleus, dorsomedial hypothalamus, and paraventricular nucleus (PVN) [2]. Analysis of transcriptional changes in the hypothalamus could potentially lead to the discovery of new elements that may modulate the response to nutrient intake.

Oligonucleotide microarrays have been used to interrogate differential expression of a large number of genes under conditions of energy excess or deficit in various tissues (reviewed by Sun [3]). Previous studies have investigated expression profiles in response to food deprivation in the whole hypothalamus [4] or in different brain nuclei or subregions [5,6]. Others have profiled gene expression in the hypothalamus of rats made obese by a high-energy diet [7,8]. We present the results of a preliminary study in which we examined differential expression of hypothalamic genes in the dietary-induced obesity model inbred mouse C57BL6 in response to a high-fat or standard chow diet. We extracted RNA from intact hypothalami and analyzed expression levels using microarrays containing ∼45 000 probes for ∼34 000 known mouse genes. Altered expression of several genes of interest was validated by quantitative real-time polymerase chain reaction (qRT-PCR).

To gain a better understanding of any underlying biological phenomena, we used Onto-Express, a tool designed to mine the available functional annotation data and find relevant biological processes [9]. It incorporates Gene Ontology (GO) project terms [10] in three structured ontologies describing gene products in terms of their associated biological processes, cellular components, and molecular functions. We input the 1511 Affymetrix probe identifications showing a difference in expression between high-fat–fed and control-fed mice. Among biological processes, the most predominant GO terms included neuropeptide signaling pathways, and among the molecular function terms, we further examined those related to neurotransmitter systems. We identified five genes involved in molecular functions related to dopamine, a critical food intake-related neurotransmitter in the hypothalamus [11], with release rapidly increased in the ventromedial nucleus during spontaneous eating [12]. Dopamine is also released in the midbrain ventral tegmental area and nucleus accumbens during consumption of food, facilitating motivational behaviors directed toward its acquisition [13,14]. We report changes in the expression of neuropeptide and neurotransmitter genes in the high-fat–fed mouse that may represent imperfect attempts to maintain energy homeostasis in the face of nutrient excess.

## Materials and methods

### Animals

Fifteen-week-old genetically unaltered female C57BL6/129SVJ (C57BL6) mice (bred in house) weighing 12.1 ± 0.7 g (mean ± standard error of the mean) at 3 wk of age were used in the study. Mice were housed in rooms maintained at 21 ± 1 °C with relative humidity at 45–65% on a 12-h light/dark cycle. Mice were fed a standard chow diet (4% fat, 6.8% kcal from fat; Rodent Diet RM1E, SDS, Essex, United Kingdom; *n* = 6) or a high-fat diet (35% fat from lard, 58.4% kcal from fat; Harlan Teklad, Madison, WI, USA, http://www.teklad.com/rodent.asp; reference TD.03584; *n* = 8). Mice were placed on the standard or high-fat diet at 3 wk of age and were maintained on either diet for 12 wk, until 15 wk of age. The mice were weighed at intervals of 2 wk from 3 to 15 wk of age (0 to 12 wk on respective diets).

The experiment was terminated at 15 wk by urethane anaesthesia with 2.5 mg/g of urethane (Sigma-Aldrich, Gillingham, United Kingdom) administered intraperitoneally followed by cervical dislocation. For each mouse, three discrete areas of adipose tissue associated with high-fat diet–induced obesity were dissected: the abdominal, mesenteric, and dorsal adipose tissues. The fat pads were then pooled for each mouse and weighed. The percentage of body weight composed of adipose tissue was evaluated after death for each mouse. After death, the skull was cut opened and the brain rotated to expose the hypothalamus. The hypothalamus was excised, placed into a 1.5-mL cryotube, snap-frozen in liquid nitrogen, and stored at −80 °C for subsequent preparation of total RNA. All procedures were carried out in accordance with the U.K. Home Office Animals (Scientific Procedures) Act, 1986, and local ethical committee guidelines.

### Analysis of results and statistical evaluation

Results are expressed as mean ± standard error of the mean. Statistical analysis was performed using SPSS (SPSS Inc., Chicago, IL, USA). Statistical evaluation was carried out using a two-way analysis of variance, followed by Bonferroni's test for comparison of means, as required by the data. *P* < 0.05 was considered statistically significant.

### RNA preparation

One milliliter of prechilled RNA*later*-ICE (Ambion, Austin, TX, USA) was added to each hypothalamus sample and incubated at −20°C for at least 16 h to prepare the frozen tissue for extraction of RNA. Total RNA was isolated from single hypothalami using the TRIzol Plus RNA purification kit (Invitrogen, Paisley, United Kingdom) according to the manufacturer's instructions. Briefly, RNA*later*-ICE was replaced with 1 mL of TRIzol, mixed to homogenize the sample, and incubated at room temperature for 5 min. The chloroform-ethanol extract was transferred to the RNA spin cartridge and centrifuged, followed by washes with buffers I and II. The RNA was eluted by adding 50 μL of RNase-free water and centrifuging and then stored at −70°C until further use. Experimental variability was minimized by batch processing individual hypothalamus samples simultaneously. Before amplification and labeling, the quality of the 14 total RNA samples was determined using the Agilent 2100 Bioanalyzer equipped with the RNA LabChip (Agilent Technologies, Palo Alto, CA, USA) according to the manufacturer's instructions. The RNA integrity number of the RNA ranged from 8.0 to 10.0. The purities of acceptable RNA samples (as measured by 260:280 absorbance ratios) were greater than 1.8 and 28S:18S ratios in the samples exceeded 1.5. All samples were therefore of sufficient quality for expression-level analysis.

### Microarray hybridization and data analysis

Seven hundred fifty nanograms of total RNA was converted to biotin-labeled cRNA using the MessageAmp II-Biotin Enhanced kit (Ambion). To minimize individual variation as a source of gene-expression variance, RNA samples were pooled, one pool representing the six control-fed mice (125 ng from each sample) and one representing the eight high-fat–fed mice (93.8 ng from each sample). Briefly, total RNA was reverse-transcribed using a T7 oligo(dT) primer to synthesize first-strand cDNA. The cDNA then underwent second-strand synthesis employing DNA polymerase and RNAse H to simultaneously degrade RNA and synthesize second-strand cDNA. After purification, the double-stranded DNA was used as a template for an in vitro transcription reaction containing biotin-modified uridine triphosphate and T7 RNA polymerase. The resulting biotin-labeled cRNA was purified to remove unincorporated nucleoside triphosphates, salts, enzymes, and inorganic phosphate.

For each cRNA, 15.5 μg was fragmented at 94°C for 35 min in fragmentation buffer (40 mM Tris-acetate, pH 8.1; 100 mM potassium acetate; 30 mM magnesium acetate). The sizes of the fragments were analyzed using the Agilent Bioanalyzer. Two hundred seventy μL of hybridization cocktail master mix was added to 30 μL of fragmented cRNA, producing 300 μL containing 50 pM control oligo B2, 1× eukaryotic hybridization controls, 0.1 mg/mL of herring sperm DNA, 0.5 mg/mL of acetylated bovine serum albumin, 1× hybridization buffer, and 10% dimethylsulfoxide. The arrays were prehybridized with 250 μL of 1× hybridization buffer for 10 min at 45°C with rotation at 60 rpm in a hybridization oven. The buffer was then removed and 200 μL of hybridization cocktail from the experimental and control samples was then hybridized to the Affymetrix Mouse Genome 430 2.0 oligonucleotide microarrays at 45°C for 16 h with rotation at 60 rpm. The arrays were then transferred to the Gene Chip Fluidics Station 400 (Affymetrix, Santa Clara, CA, USA). For each chip, 1200 μL of stain solution (1× stain buffer, 2 mg/mL of acetylated bovine serum albumin, and 0.01 mg/mL streptavidin/phycoerythrin) was prepared and aliquoted into two batches, and 600 μL of antibody solution (1× stain buffer, 2 mg/mL of acetylated bovine serum albumin, 1 mg/mL of normal goat immunoglobulin G, and 3 μg of biotinylated antibody) was prepared. The arrays were stained with stain solution, followed by antibody and finally stain solution.

Arrays were scanned for fluorescent signals using the Affymetrix GCS3000 GeneChip Scanner. After scanning, images were analyzed using Microarray Analysis Suite 5.0 algorithms with the default settings, in the GeneChip Operating Software (Affymetrix), to generate raw data in the form of CEL files. Data were globally scaled to correct for any small variations in hybridization efficiency, laser power, etc., and comparison analyses were set up in GeneChip Operating Software. We first made a preliminary selection that removed all probe sets for which control- and high-fat–fed samples produced an “absent” call (detection *P* ≥ 0.065), according to Affymetrix's test for the presence of a transcript, followed by removal of “no-change” calls, where no significant difference was observed between control- and high-fat–fed samples (change *P* = 0.002667 to 0.997333).

### Analysis of gene expression by qRT-PCR

Quantitative RT-PCR was used to confirm the microarray results for the expression levels of seven genes. Total RNA from three control- and three high-fat–fed mouse hypothalamic RNA samples was first reverse-transcribed into cDNA using the Enhanced Avian RT First-Strand Synthesis Kit (Sigma, St. Louis, MO, USA) in a 20-μL reaction containing 1 μg of RNA and random nonamer primers. Control reactions, omitting reverse transcriptase, were also performed. Sensitive, high-throughput assays were designed, optimized, and run for each gene using the SYBR Green JumpStart Taq ReadyMix (Sigma) and the ABI 7000 sequence detector (Applied Biosystems, Foster City, CA, USA). Each 25-μL reaction contained approximately 100 pg of cDNA and 200 μM of each primer. Primers were designed by Sigma, applied to mouse cDNA sequences derived from the National Center for Biotechnology Information website (http://www.ncbi.nlm.nih.gov), and are listed in Table 1. Primers were tested with (non-quantitative) PCR to optimize reaction conditions to generate a single PCR product, as shown by gel electrophoresis. Primer sets producing multiple bands were discarded and redesigned. Ribosomal protein L19 (*Rpl19*) was used as a housekeeping gene. An identical cycle profile was used for all genes: 94°C for 2 min + (94°C for 15 s + 60°C for 1 min + 72°C for 1 min) × 45 cycles followed by a dissociation stage. Gene-expression levels were calculated with the relative quantitation method [15] and normalized to the *Rpl19* level.

All PCR reactions were carried out in duplicate. To make accurate comparisons among samples and adjust target gene data for the control gene *Rpl19*, the efficiency of the two PCR reactions must be similar. Dilutions of cDNA were run in triplicate using the optimal primer concentrations. The average threshold cycle (Ct) value for each dilution was plotted against the log of the input cDNA amount to evaluate linearity of the assay. To find the relative efficiency, the difference between average target gene Ct and average control gene Ct (▵Ct) for each dilution was plotted against the log-input cDNA amounts. A slope with an absolute value <0.1 indicates that the efficiencies of the target gene and control gene reactions are similar, allowing the comparative Ct method to be used for relative quantification. In validation experiments, each assay found to be linear over three dilutions. The absolute values of the slope for each comparison were <0.1, indicating equal efficiencies and specificity for targets. Thus the *Rp119* assay could be used to accurately adjust target gene data. The qRT-PCR analyses were performed on RNA samples from three standard-fed and three high-fat–fed mice. The mean fold change of expression in high-fat–fed mice compared with standard-fed mice was calculated using the 2^−ΔΔCt^ method [16] and the range of the fold changes was calculated from the standard error of the ΔΔCt values.

### Bioinformatics

To perform an analysis of GO functional categories according to the Gene Ontology Consortium (http://www.geneontology.org), we used a freely available software package Onto-Express (available at http://vortex.cs.wayne.edu/projects.htm#Onto-Express) [9]. Onto-Express searches the public databases and constructs functional profiles using GO terms [10]. We searched the following categories: cellular component, biological process, and molecular function. The result of these analyses is a functional profile of the condition studied, accompanied by the computation of significance values for each functional category. Significant biological processes are distinguished from random events by comparing the actual number of occurrences with the expected number for each category.

## Results

### Phenotypic changes in high-fat–fed mice compared with controls

**Fig. 1:**
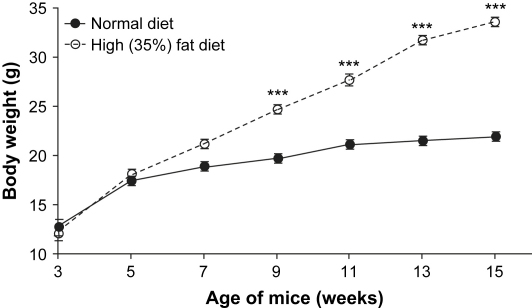
Change in body weight of female C57BL6 mice. Mice were fed a normal (*n* = 6) or high-fat (35%, *n* = 8) diet from 0 to 12 wk (3 to 15 wk of age). ^∗∗∗^ Statistical evaluation of mean ± SEM, *P* < 0.001 by two-way analysis of variance with Bonferroni's correction.

The mice gained weight from 3 to 15 wk, as shown in Figure 1. The mice on the standard diet exhibited a stable weight from 11 wk of age; those on the 35% high-fat diet continued to gain weight throughout the experimental period. The mean weight ± standard error of the mean of the mice at 3 wk of age was 12.1 ± 0.7 g and increased to 21.5 ± 0.39 g (*n* = 6) on the standard diet (gain 9.4 g) and 33.9 ± 0.75 g (*n* = 8) on the high-fat diet (gain 21.8 g) by 15 wk of age. High-fat–fed mice therefore gained 2.32 times more weight in 12 wk than their standard-fed littermates. The differences in mean weights between high-fat- and standard-fed mice were highly significant (*P* < 0.001) from age 9 wk onward (6 wk on diets). In these mice, the percentage of body weight comprised of adipose tissue in the three pooled major fat pads was significantly different at termination: 2.4 ± 0.3% (*n* = 6) for mice fed a standard diet and 16.6 ± 0.9% (*n* = 8) for mice fed the high-fat diet (*P* < 0.0001).

### Microarray analysis

After removing all probe sets on the Affymetrix Mouse Genome 430 2.0 gene chip array for which control- and high-fat–fed samples produced an “absent” or “no-change” call, 1511 probe sets indicated a difference in gene expression between high-fat–fed and control-fed mice. The identifications of the 1511 probe sets and the .CEL level data (probe cell data) are available as an online supplement (Online Table 1). These probes represented 1212 unique gene transcripts with altered expression, of which 616 (50.8%) were upregulated and 596 (49.2%) were downregulated. The full results, showing the 1212 unique gene transcripts and fold changes in expression in high-fat–fed compared with control-fed mice, are available as an online supplement (Online Table 2). Tables 2 and 3 present the genes on this list, with the greatest changes in expression in response to the high-fat diet. There were 45 genes with expression increased by 2.0-fold or more in high-fat–fed mice compared with controls (Table 2) and 38 genes with expression decreased by 2.0-fold or more, i.e., to less than 50% of the control levels (Table 3).

### Gene ontology

**Fig. 2:**
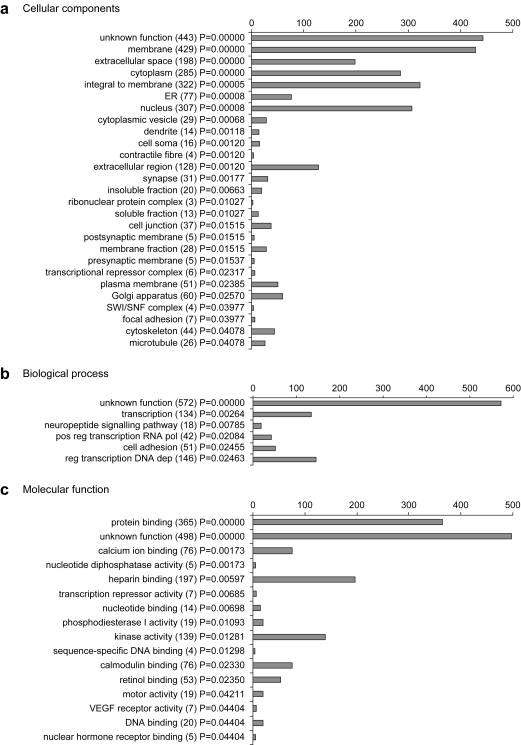
Change in gene expression by high-fat feeding. Significant representation in Gene Ontology 1511 probes indicated a difference in gene expression between high-fat–fed (*n* = 8) and control-fed (*n* = 6) mice. This subset was processed using Onto-Express to categorize the genes according to (a) biological process, (b) cellular component, and (c) molecular function. In each group, significant functions were distinguished from random events by comparing the actual number of occurrences with the expected number for each Gene Ontology term, predicted by probe representation on the microarray. The Gene Ontology terms significantly represented (*P* < 0.05 in left column) are shown. The length of each bar is proportional to the number of identifiers found in the input file (probe identifications) that were annotated using the corresponding term. The numbers of probe identifications in parentheses and *P* values for each term are shown next to each bar. ER, endoplasmic reticulum; SWI/SNF, SWItch/Sucrose Nonfermentable; VEGF, Vascular endothelial growth factor.

To gain a better understanding of any underlying biological phenomena, we used Onto-Express [9], a tool designed to mine the available functional annotation data and find relevant biological processes (see materials and methods). It incorporates GO project terms [10] in three structured ontologies that describe gene products in terms of their associated biological processes, cellular components, and molecular functions. We input the 1511 Affymetrix probe identifications showing a difference in gene expression between high-fat–fed and control-fed mice. The significance of gene representation in each GO term is computed from the number of regulated genes and the number of genes on the microarray. The tree view displays the results in the same format as the ontology from the Gene Ontology Consortium. Expansion of the tree allows identification of the individual genes in each significant GO term. An overview is provided by the tabulated output for GO terms in various functional categories. GO terms with significant representation in three categories, 1) cellular component, 2) biological process, and 3) molecular function, are shown in Figure 2.

Among cellular components, the most significantly represented were cell membrane, extracellular space, and cytoplasm, structures integral to the membrane, the endoplasmic reticulum, and nucleus (all *P*s < 0.0001). Among biological processes, the most predominant related to transcription and neuropeptide signaling pathways (*P* < 0.001). Less significant were regulation of transcription and cell adhesion (*P* < 0.01). Among molecular functions, many types of binding activity featured the more widespread, calcium ion, nucleotide, DNA, and nuclear hormone receptor, and the more specific, heparin, calmodulin, and retinol. Various enzyme activities were represented, including nucleotide diphosphatase, phosphodiesterase I and kinase, and transcriptional repressors (*P*s = 0.0017 to 0.044).

Expansion of the tree view allows further interrogation of GO terms. Among biological processes, we found significant representation in neuropeptide signaling pathways. Among molecular functions, we investigated GO terms linked to receptors, synthetic enzymes, and transporters of cholinergic, dopaminergic, adrenergic, γ-aminobutyric acidergic, glutamatergic, or serotonergic neurotransmitter systems.

### Genes involved in neuropeptide signaling

Four index genes in the neuropeptide signaling pathway (GO:0007218), with well-documented evidence of a role in energy homeostasis, showed predicted alterations in expression in response to excess nutrient intake. Orexigenic *Agrp*, agouti-related polypeptide, was downregulated 0.33-fold (33% of standard-fed levels) by high-fat feeding and *Npy*, neuropeptide Y, 0.57-fold. Anorexigenic *Cart*, cocaine and amphetamine-regulated transcript prepropeptide, was upregulated 1.3-fold (30% above standard fed) and *Pomc*, proopiomelanocortin, 1.4-fold.

### Genes involved in neurotransmission

We identified five genes involved in molecular functions related to dopamine. Two were involved with production and release: *Th*, tyrosine hydroxylase (up 1.3-fold), catalyses the rate-limiting biosynthetic step in dopamine synthesis from tyrosine (GO:0006585) and *Slc18a2*, solute carrier family 18 (vesicular monoamine), member 2 (up 1.2-fold), is involved in dopamine transmembrane transporter activity (GO:0005275). Three were involved with reuptake, regulating transmission, and degradation: *Slc6a3*, the dopamine/sodium symporter, is responsible for dopamine uptake (GO:0005330, up 3.48-fold), *Snca* α-synuclein (up 1.1-fold) is a negative regulator of dopamine neurotransmission (GO:00042416), and *Maoa* monoamine oxidase (up 1.2-fold) is involved in dopamine deamination (GO:0042420).

A number of other regulated genes were involved with different neurotransmitter systems. *Viaat*, solute carrier family 32 (γ-aminobutyric acid vesicular transporter), member 1, which has γ-aminobutyric acid/hydrogen symport activity (GO:0015495), was downregulated (0.70-fold), as was *Atp2b2* adenose triphosphatase, Ca^2+^ transporting, plasma membrane 2 (0.62-fold), involved in the serotonin metabolic process (GO:0042428). *Chrna6* (up 2.3-fold) is involved in cholinergic synaptic transmission (GO:0005216) and in regulation of dopamine secretion (GO:0014059). *Grm3* and *Grm8*, glutamate receptors metabotropic 3 and 8 (both up 1.3-fold), and *Cacnb4* calcium channel, voltage-dependent, β4 subunit (up 1.1-fold), are involved in glutamatergic transmission (GO:0035249).

### Confirmation of microarray data by qRT-PCR analysis

**Fig. 3:**
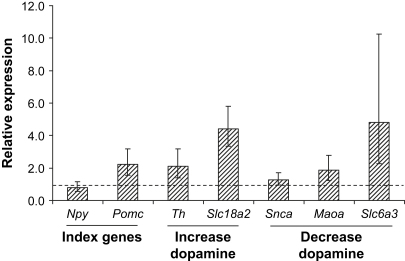
Validation of microarray data by quantitative real-time polymerase chain reaction. The average level of expression of each gene after the high-fat diet normalized to *Rpl19* and relative to expression on the normal diet (dashed line) is shown. The error was estimated by evaluating the 2^−ΔΔCt^ term using ΔΔCt ± SD, producing a range of values asymmetrically distributed relative to the average value, shown by the bars. *Maoa*, monoamine oxidase; *Npy*, neuropeptide Y; *Pomc*, pro-opiomelanocortin; *Slc18a2*, solute carrier family 18 (vesicular monoamine), member 2 (vesicular monoamine transporter-2); *Slc6a3*, solute carrier family 6 (neurotransmitter transporter, dopamine), member 3; *Snca*, α-synuclein; *Th*, tyrosine hydroxylase.

To validate the microarray analyses of the five genes involved in dopamine availability, *Th*, *Slc18a2, Slc6a3*, *Snca*, and *Maoa*, we examined the mRNA levels of these and two index genes *Pomc* and *Npy* by qRT-PCR. The results of analysis of mRNAs isolated from hypothalami of three high-fat–fed and three control-fed mice are shown in Figure 3. The mRNA levels established by qRT-PCR confirmed the direction of the changes indicated on the arrays and showed higher actual fold changes than indicated by the microarray data. The fold changes in high-fat–fed mice compared with controls were as follows: *Npy* 0.80; *Pomc* 2.22; *Th* 2.10; *Slc18a2* 4.40; *Snca* 1.28; *Maoa* 1.86; and *Slc6a3* 4.82.

## Discussion

The hypothalamus maintains energy homeostasis by integrating signals from peripheral sites and producing signaling molecules in response to changes in energy status [2,17]. In this preliminary study, we aimed to obtain a broad overview of the transcriptional changes that accompany this complex process and possibly identify novel players, using microarrays to discover genes whose expression in mice is altered in response to a high-fat diet. We identified 1212 genes with altered expression. Regulation of four neuropeptide genes and five involved in dopaminergic transmission emerged from the analysis of gene ontology.

We used single arrays for pooled RNA samples from high-fat- or control-fed mice, which avoided variability in gene-expression patterns between samples. In a complex action such as energy balance, numerous genes with small to moderate effects are expected to contribute to variation, with changes in vivo unlikely to exceed three-fold [4] and considerable variation expected between genes. In mice on the high-fat diet, we detected increases greater than two-fold in 45 genes and 38 genes were downregulated to less than 50% of control levels. We chose to analyze all probes signifying altered expression using Onto-Express software without imposing a cutoff, so that any showing small yet functionally significant changes would not be excluded at the outset. We confirmed changes in expression of seven genes in response to high-fat feeding by qRT-PCR, suggesting that differences in levels of mRNA are correlated to diet and the genes are likely downstream targets of regulation by nutritional state. The mRNA levels of each gene showed modest variation among the three mice, with the exception of *Slc6a3*, where a divergent response in mice fed the high-fat diet led to a large statistical range in the mean fold changes. *Slc18a2* showed considerably higher relative expression in the qRT-PCR than was evident from the microarray data.

Two physiologically opposing populations of ARC neurones are crucial to energy homeostasis. The first is located predominantly in the ventromedial ARC and coexpresses the orexigenic neuropeptides neuropeptide Y and agouti-related protein. The second is located predominantly in the ventrolateral ARC and coexpresses anorexigenic neuropeptides derived from the pro-opiomelanocortin prohormone (e.g., α-melanocyte–stimulating hormone) and the cocaine- and amphetamine-regulated transcript. Neural fibers from both ARC regions project to the paraventricular nucleus, the site of second-order neuronal signaling [2]. At least two research groups have microdissected the hypothalamus to examine gene expression specifically in the ARC [7] or ARC and paraventricular nucleus [18,19] in relation to nutrient intake. This demands exceptional skill and the quantitation of RNA in very small amounts of material. However, the difficulty of excision without contamination from adjacent areas means that these dissections can still be relatively heterogeneous at the cellular level. In common with a number of other investigators examining the response of hypothalamic genes to the nutritional state [4,20,21], we dissected intact hypothalamus from the brain. We concede that, in processing the whole tissue, the magnitude of changes that might have been observed in specific nuclei was inevitably reduced and any opposing effects in different nuclei would not be seen.

The adipocyte hormone leptin signals levels of peripheral fat stores to the ARC of the ventral hypothalamus [22]. Most previous reports of *Pomc*, *Cart*, *Npy*, and *Agrp* expression after dietary manipulation have shown a change contrary to the maintenance of homeostasis or no change in obesity-prone mice. For example, leptin excites the anorexigenic *Pomc*- and *Cart*-expressing neurons, reducing appetite and enhancing metabolism [23]. However, in the diet-induced obese mouse, *Pomc* and *Cart* have shown downregulation [24–26], no change [27,28], and upregulation [29] on a high-fat diet. Orexigenic *Npy* and *Agrp* production is inhibited by leptin, so downregulation is the expected response. In DIO mice elevated *Npy* expression is usual [27,30–36] and has been cited as the reason for obesity susceptibility, but downregulation [27,29,37–39] or no change [28,30,40] has also been reported. It therefore appears that varying degrees of homeostatic efficiency by neuropeptides exist in the obesity-prone mouse model. Our microarrays showed changes in line with a normal homeostatic response to nutrient excess: increased expression of *Pomc* and *Cart* and decreases in *Npy* and *Agrp* after 12 wk on the high-fat diet. However, homeostasis was imperfect because the mice gained more than twice as much weight, attributable to significantly more adipose tissue, than those on standard chow. Failure of the mice to control body weight must result from an inability of the changes in hypothalamic neuropeptide expression alone to effectively counteract the obesogenic drive of the high-fat intake mediated by other factors.

To identify novel genes involved in response to the high-fat diet, we focused on receptors, synthetic enzymes and transporters of cholinergic, dopaminergic, adrenergic, γ-aminobutyric acidergic, glutamatergic, or serotonergic neurotransmitter systems, identified through analysis of gene ontology. A group of five genes with altered expression was directly related to the activity of dopamine. *Th* is the gene for tyrosine hydroxylase, the rate-limiting enzyme in dopamine biosynthesis [41]. *Slc18a2* is the gene for solute carrier family 18, member A2 (vesicular monoamine transporter-2), the only transporter that moves cytoplasmic dopamine into synaptic vesicles for storage and subsequent exocytotic release [42]. Together, these genes control dopamine production and exocytosis. Upregulation of the other three genes reduces dopamine availability. *Snca* is the gene for α-synuclein, involved in the regulation of dopamine biosynthesis [43], release, and transport [44]. *Slc6a3* is the gene for solute carrier family 6, member A3 (neurotransmitter transporter, dopamine), which terminates the action of dopamine by its high affinity sodium-dependent reuptake into presynaptic terminals [45]. *Maoa*, the gene for monoamine oxidase, deaminates dopamine [46].

Dopamine signals by five G-protein–coupled receptor subtypes comprising two families. The D1-like receptors DRD1 and DRD5 activate adenylyl cyclase and the D2-like receptors DRD2, DRD3, and DRD4 inhibit the enzyme in postsynaptic neurons [47]. Drugs that block DRD2 enhance appetite and induce weight gain in animals and humans [48]. DRD2 and DRD1 agonists have the opposite effect [49]. The effects of changing dopamine concentration in the hypothalamus and midbrain on feeding behavior are well known. Dopamine release increases rapidly in the ventromedial nucleus during spontaneous eating [12] and food deprivation brings about an immediate decrease [11]. Dopamine levels are elevated in the brains of obese animal models [12,50], but at the same time, DRD2 expression is decreased [51]. This has given rise to the idea that enhanced dopamine release may compensate for the smaller number of receptors to maintain signal transduction. Food consumption is a rewarding and highly reinforcing behavior that not only provides nutrients needed for survival but also induces feelings of gratification [52]. Midbrain dopamine neurons have long been known to mediate reward behavior and the motivational aspects of feeding [13,14]. The reward pathway includes the dopamine neurons in the ventral tegmental area that project to the nucleus accumbens, and dopamine release in the rat nucleus accumbens has been shown to relate to the amount of food ingested [53]. Lack of DRD2 receptors transducing an appetite-reducing or satiety signal has been proposed to induce a “reward deficiency syndrome” [54] in which increased release of dopamine in response to large meals in obese animals compensates for reduced signaling capability and, hence, its reduced feedback effect on satiety [51]. Geiger et al. [55] hypothesized that increased food intake in underweight animals could be partly attributed to a compensatory attempt to restore basal mesolimbic dopamine levels. They showed that extracellular basal dopamine is low in obesity-prone rats due to reduced activity of tyrosine hydroxylase and vesicular monoamine transporter-2 and attributed susceptibility to obesity to an increased motivation to eat and increase dopamine levels.

Our obesity-prone mice gained more weight on the high-fat than on the standard diet, but we found no change in expression of dopamine receptor genes, only those influencing the availability of dopamine. The net effect of altered expression of the five genes on extracellular dopamine concentration cannot be predicted at present because no measurements were made. An increase would be compatible with palatable high-fat food evoking a greater surge in dopamine than the less palatable chow [12], i.e., providing a greater reward, which would encourage overeating. We cannot comment directly on any stimulatory effect of the palatable high-fat diet because we did not measure food intake. However, Chan et al. [56] reported a significant increase in C57BL6 mice on a high-fat diet (21%) compared with a standard diet after 4 and 10 wk, suggesting that changes in gene expression in our mice may have been evoked by overnutrition. It would require a pair-feeding experiment with caloric intake matched in high-fat–fed and chow-fed controls to establish whether fat as a dietary constituent or overnutrition per se was the critical factor in eliciting the observed changes in gene expression.

Mechanisms by which high-fat feeding or caloric excess could upregulate these genes await further investigation. The changes may be linked to leptin action because the mice remained sensitive enough for the normal homeostatic changes in hypothalamic neuropeptides to be evoked. In addition to its anorexigenic effects in the hypothalamus, leptin inhibits the firing of dopamine neurons in the ventral tegmental area of the midbrain, leading to a decrease in dopamine release [57] and reduction in reward-driven feeding [58,59]. Alternatively, the genes may respond directly to circulating nutrients through activation and binding of transcription factor complexes.

It should be noted that, in addition to its influence on food intake, dopamine has a potential role in energy balance through its involvement in thermogenesis. Cold exposure activates tyrosine hydroxylase, producing a large increase in dopamine content and turnover rate in brown adipose tissue [60]. In contrast, activation of central nervous system dopamine DRD2 receptors inhibits sympathetically mediated interscapular brown adipose tissue thermogenesis in response to cold exposure, inducing hypothermia [61]. Therefore, central dopamine acting on DRD2 receptors in the hypothalamus appears to inhibit thermogenesis and appetite without apparent overall directional effect on energy balance.

Obesity is a leading public health issue, categorized by the World Health Organization as a global epidemic and driven by the current environment of overabundance of food and lack of physical activity. Obesity and overweight are major risk factors for chronic diseases, including type 2 diabetes, cardiovascular disease, and certain cancers. It is imperative to understand the mechanisms underlying obesity to devise prevention and treatment strategies. All known monogenic defects causing human obesity disrupt hypothalamic pathways, affecting satiety and food intake rather than influencing metabolic rate or deposition of fat. It is likely that genes contributing to common human obesity will have a similar function. In an environment with few restraints on food availability, homeostatic responses to overnutrition mediated by neuropeptides designed to protect from starvation may be less relevant to targeting the problem of obesity than pathways involving the hedonic response to food. The involvement of the dopamine system in reward and reinforcement has led to the hypothesis that low brain dopamine activity in obese subjects predisposes to excessive eating, as a means to compensate for decreased activation of these circuits. Strategies aimed at improving dopamine function have been shown to be beneficial in the treatment of obese individuals. This may prove to be a more productive line of investigation in the quest to solve an increasingly important public health issue in Western societies.

## Conclusion

After a high-fat diet, mouse hypothalamic genes for synthesis and release of dopamine were upregulated, as were those for uptake and degradation, but there was no change in expression of receptors. Failure to counter the rewarding effects of dopamine released by overfeeding could contribute to diet-induced obesity in these mice.

## Figures and Tables

**Table 1:** Primer sequences for gene expression analysis by quantitative real-time polymerase chain reaction

Gene	Forward (5′–3′)	Reverse (5′–3′)	Size (bp)	Accession no.
*Pomc*	AGAACGCCATCATCAAGAAC	AAGAGGCTAGAGGTCATCAG	109	NM_008895
*Npy*	GTGGATCTCTTCTCTCACAGAGG	GCCCAAACACACGAGCAGAG	143	NM_023456
*Maoa*	GGAGAAGCCCAGTATCACAGG	GAACCAAGACATTAATTTTGTATTCTGAC	113	NM_173740
*Slc6a3*	GCATCCTGTTCACATATTACAC	TTGTCTCCCAACCTGAATTC	144	NM_010020
*Slc18a2*	TGATCCTGTTCATCGTGTTCCTC	GCTGGGAATGATGGGAACTACG	77	NM_172523
*Snca*	TGTACAGTGTGTTTCAAAGTCTTCC	GAAGCCACAACAATATCCACAGC	129	NM_001042451
*Th*	CCCCACCTGGAGTACTTTGTG	CTTGTCCTCTCTGGCACTGC	111	NM_009377

*Maoa*, monoamine oxidase; *Npy*, neuropeptide Y; *Pomc*, pro-opiomelanocortin; *Slc18a2*, solute carrier family 18 (vesicular monoamine), member 2 (vesicular monoamine transporter-2); *Slc6a3*, solute carrier family 6 (neurotransmitter transporter, dopamine), member 3; *Snca*, α-synuclein; *Th*, tyrosine hydroxylase.

**Table 2:** Genes with expression increased after high-fat diet by at least two-fold compared with standard diet∗

Probe identification	Fold change	Gene symbol	Gene name
1418047_at	13.00	*Neurod6*	Neurogenic differentiation-6
1434802_s_at	6.06	*Ntf3*	Neurotrophin-3
1419230_at	5.28	*Krt1-12*	RIKEN cDNA A830036E02 gene
1445228_at	4.59	*Spred1*	Sprouty protein with EVH-1 domain 1, related sequence
1417415_at	3.48	*Slc6a3*	Solute carrier family 6 (neurotransmitter transporter, dopamine), member 3
1424649_a_at	3.25	*Tm4sf3*	Tetraspanin-8
1441107_at	3.03	*Dmrta2*	Doublesex and mab-3 related transcription factor-like family A2
1446627_at	3.03	*Stk3*	Serine/threonine kinase-3 (Ste20, yeast homolog)
1445555_at	2.83	*Trpm3*	Transient receptor potential cation channel, subfamily M, member 3
1418271_at	2.64	*Bhlhb5*	Basic helix-loop-helix domain containing, class B5
1421944_a_at	2.64	*Asgr1*	Asialoglycoprotein receptor 1
1445552_at	2.64	*Mlr1*	Ligand dependent nuclear receptor corepressor-like
1447869_x_at	2.64	*Rhobtb3*	Rho-related BTB domain containing 3
1448265_x_at	2.64	*Eva1*	Epithelial V-like antigen 1
1451191_at	2.64	*Crabp2*	Cellular retinoic acid binding protein II
1417072_at	2.46	*Slc22a6*	Solute carrier family 22 (organic anion transporter), member 6
1424679_at	2.46	*Mab21l1*	Mab-21–like 1 (*Clostridium elegans*)
1428986_at	2.46	*Slc17a7*	Solute carrier family 17 (sodium-dependent inorganic phosphate cotransporter), member 7
1430460_at	2.46	*5830410F13Rik*	RIKEN cDNA 2310047O13 gene
1437244_at	2.46	*Gas2l3*	Growth arrest-specific 2 like 3
1440488_at	2.46	*Ptpn4*	Protein tyrosine phosphatase, non-receptor type 4
1447567_at	2.46	*Odz3*	Odd Oz/ten-m homolog-3 (*Drosophila*)
1457656_s_at	2.46	*C230085N15Rik*	RIKEN cDNA C230085N15 gene
1419662_at	2.30	*Ogn*	Osteoglycin
1443012_at	2.30	*Tcf12*	Transcription factor 12
1449254_at	2.30	*Spp1*	Secreted phosphoprotein 1
1450427_at	2.30	*Chrna6*	Cholinergic receptor, nicotinic, α-polypeptide 6
1452473_at	2.30	*E130201N16Rik*	Proline rich 15
1416236_a_at	2.14	*Eva1*	Epithelial V-like antigen 1
1426852_x_at	2.14	*Nov*	Nephroblastoma overexpressed gene
1442785_at	2.14	*A130004G11Rik*	RIKEN cDNA A130004G11 gene
1443131_at	2.14	*Lrp1b*	Low-density lipoprotein–related protein 1B (deleted in tumors)
1443337_at	2.14	*B130020M22Rik*	RIKEN cDNA B130020M22 gene
1443783_x_at	2.14	*H2-Aa*	Histocompatibility 2, class II antigen A, α
1459369_at	2.14	*Epha6*	Eph receptor A6
1419663_at	2.00	*Ogn*	Osteoglycin
1419665_a_at	2.00	*Nupr1*	Nuclear protein 1
1421477_at	2.00	*Cplx2*	Complexin 2
1426851_a_at	2.00	*Nov*	Nephroblastoma overexpressed gene
1429537_at	2.00	*5730406M06Rik*	RIKEN cDNA 5730406M06 gene
1430770_at	2.00	*3110080E11Rik*	RIKEN cDNA 3110080E11 gene
1431248_at	2.00	*5031426D15Rik*	RIKEN cDNA 5031426D15 gene
1435290_x_at	2.00	*H2-Aa*	Histocompatibility 2, class II antigen A, α
1444152_at	2.00	*Cugbp2*	CUG triplet repeat, RNA binding protein 2
1457555_at	2.00	*Gpr151*	G-protein–coupled receptor 151
1458263_at	2.00	*Cugbp2*	CUG triplet repeat, RNA binding protein 2

∗ Two-fold signifies expression after high-fat diet is increased to 200% level after the standard diet.

**Table 3:** Genes with expression decreased after high-fat diet by ≥0.5-fold compared with standard diet∗

Probe identification	Fold change	Gene symbol	Gene name
1417109_at	0.50	*Lcn7*	Tubulointerstitial nephritis antigen-like
1422410_at	0.50	*Ferd3l*	Fer3-like (*Drosophila*)
1423424_at	0.50	*Zic3*	Zinc finger protein of the cerebellum 3
1424272_at	0.50	*Stat3*	Signal transducer and activator of transcription 3
1434574_at	0.50	*9430008C03Rik*	RIKEN cDNA 9430008C03 gene
1435370_a_at	0.50	*Ces3*	Carboxylesterase 3
1438086_at	0.50	*Npy6r*	Neuropeptide Y receptor Y6
1440519_at	0.50	*Sp8*	Trans-acting transcription factor 8
1448890_at	0.50	*Klf2*	Kruppel-like factor 2 (lung)
1454608_x_at	0.50	*Ttr*	Transthyretin
1455913_x_at	0.50	*Ttr*	Transthyretin
1459737_s_at	0.50	*Ttr*	Transthyretin
1419300_at	0.47	*Flt1*	FMS-like tyrosine kinase 1
1428664_at	0.47	*Vip*	Vasoactive intestinal polypeptide
1439627_at	0.47	*Zic1*	Zinc finger protein of the cerebellum 1
1457094_at	0.47	*She*	src homology 2 domain-containing transforming protein E
1426732_at	0.44	*Des*	Desmin
1441924_x_at	0.44	*Edn3*	Endothelin 3
1451617_at	0.44	*Rho*	Rhodopsin
1440926_at	0.41	*Flt1*	FMS-like tyrosine kinase 1
1450371_at	0.41	*Tshb*	Thyroid-stimulating hormone, β subunit
1439427_at	0.38	*Cldn9*	Claudin 9
1424107_at	0.35	*Kif18a*	Kinesin family member 18A
1421690_s_at	0.33	*Agrp*	Agouti related protein
1450293_at	0.33	*Mageb3*	Melanoma antigen, family B, member 3
1419974_at	0.31	*Scp2*	Sterol carrier protein 2, liver
1424794_at	0.31	*9130020G10Rik*	Ring finger protein 186
1446059_at	0.31	*2900086E13Rik*	RIKEN cDNA 2900086E13 gene
1436835_at	0.23	*Crk*	v-crk sarcoma virus CT10 oncogene homolog (avian)
1438737_at	0.20	*Zic3*	Zinc finger protein of the cerebellum 3
1459356_at	0.15	*Dst*	Dystonin
1445657_at	0.13	*AW111846*	Expressed sequence AW111846
1427727_x_at	0.13	*Psg19*	Pregnancy-specific glycoprotein 19
1441896_x_at	0.11	*Obfc1*	Oligonucleotide/oligosaccharide-binding fold containing 1
1441650_at	0.09	*Arhgap15*	Rho GTPase activating protein 15
1427303_at	0.09	*Enpp3*	Ectonucleotide pyrophosphatase/phosphodiesterase 3
1458004_at	0.07	*Prrx1*	Paired related homeobox 1
1449844_at	0.06	*Slco1a1*	Solute carrier organic anion transporter family, member 1a1
1420112_at	0.05	*Pacs1*	Phosphofurin acidic cluster sorting protein 1
1441504_at	0.04	*Gm817*	Gene model 817 (NCBI)
1442433_at	0.04	*Pcna*	Proliferating cell nuclear antigen
1459570_at	0.02	*Gpr143*	G-protein–coupled receptor 143

∗ 0.5-fold signifies expression after high-fat diet is reduced to 50% of level after the standard diet.

## References

[1] Lam T.K., Schwartz G.J., Rossetti L. (2005). Hypothalamic sensing of fatty acids. Nat Neurosci.

[2] Schwartz M.W., Woods S.C., Porte D., Seeley R.J., Baskin D.G. (2000). Central nervous system control of food intake. Nature.

[3] Sun G. (2007). Application of DNA microarrays in the study of human obesity and type 2 diabetes. OMICS.

[4] Mobbs C.V., Yen K., Mastaitis J., Nguyen H., Watson E., Wurmbach E. (2004). Mining microarrays for metabolic meaning: nutritional regulation of hypothalamic gene expression. Neurochem Res.

[5] Zhao X., Lein E.S., He A., Smith S.C., Aston C., Gage F.H. (2001). Transcriptional profiling reveals strict boundaries between hippocampal subregions. J Comp Neurol.

[6] Bonaventure P., Guo H., Tian B., Liu X., Bittner A., Roland B. (2002). Nuclei and subnuclei gene expression profiling in mammalian brain. Brain Res.

[7] Middleton F.A., Ramos E.J., Xu Y., Diab H., Zhao X., Das U.N. (2004). Application of genomic technologies: DNA microarrays and metabolic profiling of obesity in the hypothalamus and in subcutaneous fat. Nutrition.

[8] De Souza C.T., Araujo E.P., Bordin S., Ashimine R., Zollner R.L., Boschero A.C. (2005). Consumption of a fat-rich diet activates a proinflammatory response and induces insulin resistance in the hypothalamus. Endocrinology.

[9] Draghici S., Khatri P., Martins R.P., Ostermeier G.C., Krawetz S.A. (2003). Global functional profiling of gene expression. Genomics.

[10] Ashburner M., Ball C.A., Blake J.A., Botstein D., Butler H., Cherry J.M. (2000). Gene ontology: tool for the unification of biology. The Gene Ontology Consortium. Nat Genet.

[11] Meguid M.M., Fetissov S.O., Blaha V., Yang Z.J. (2000). Dopamine and serotonin VMN release is related to feeding status in obese and lean Zucker rats. Neuroreport.

[12] Orosco M., Rouch C., Meile M.J., Nicolaidis S. (1995). Spontaneous feeding-related monoamine changes in rostromedial hypothalamus of the obese Zucker rat: a microdialysis study. Physiol Behav.

[13] Martel P., Fantino M. (1996). Mesolimbic dopaminergic system activity as a function of food reward: a microdialysis study. Pharmacol Biochem Behav.

[14] Kelley A.E., Baldo B.A., Pratt W.E., Will M.J. (2005). Corticostriatal-hypothalamic circuitry and food motivation: integration of energy, action and reward. Physiol Behav.

[15] Bustin S.A. (2000). Absolute quantification of mRNA using real-time reverse transcription polymerase chain reactions assays. J Mol Endocrinol.

[16] Livak K.J., Schmittgen T.D. (2001). Analysis of relative gene expression data using real-time quantitative PCR and the 2(-Delta Delta C(T)) method. Methods.

[17] Kalra S.P., Dube M.G., Pu S., Xu B., Horvarth T.L., Kalra P.S. (1999). Interacting appetite-regulating pathways in the hypothalamic regulation of body weight. Endocr Rev.

[18] Li J.Y., Lescure P.A., Misek D.E., Lai Y.M., Chai B.X., Kuick R. (2002). Food deprivation- induced expression of minoxidil sulfotransferase in the hypothalamus uncovered by microarray analysis. J Biol Chem.

[19] Li J.Y., Kuick R., Thompson R.C., Misek D.E., Lai Y.M., Liu Y.Q. (2005). Arcuate nucleus transcriptome profiling identifies ankyrin repeat and suppressor of cytokine signalling box-containing protein 4 as a gene regulated by fasting in central nervous system feeding circuits. J Neuroendocrinol.

[20] Heijboer A.C., Voshol P.J., Donga E., van Eden C.G., Havekes L.M., Romijn J.A. (2005). High fat diet induced hepatic insulin resistance is not related to changes in hypothalamic mRNA expression of NPY, AgRP, POMC and CART in mice. Peptides.

[21] Bullen J.W., Ziotopoulou M., Ungsunan L., Misra J., Alevizos I., Kokkotou E. (2004). Short-term resistance to diet-induced obesity in A/J mice is not associated with regulation of hypothalamic neuropeptides. Am J Physiol Endocrinol Metab.

[22] Frederich R.C., Lollmann B., Hamann A., Napolitano-Rosen A., Kahn B.B., Lowell B.B. (1995). Expression of ob mRNA and its encoded protein in rodents. Impact of nutrition and obesity. J Clin Invest.

[23] Cowley M.A., Smart J.L., Rubinstein M., Cerdan M.G., Diano S., Horvath T.L. (2001). Leptin activates anorexigenic POMC neurons through a neural network in the arcuate nucleus. Nature.

[24] Huang X.F., Han M., South T., Storlien L. (2003). Altered levels of POMC, AgRP and MC4-R mRNA expression in the hypothalamus and other parts of the limbic system of mice prone or resistant to chronic high-energy diet-induced obesity. Brain Res.

[25] Tian D.R., Li X.D., Shi Y.S., Wan Y., Wang X.M., Chang J.K. (2004). Changes of hypothalamic alpha-MSH and CART peptide expression in diet-induced obese rats. Peptides.

[26] Yu Y., South T., Wang Q., Huang X.F. (2008). Differential expression of hypothalamic CART mRNA in response to body weight change following different dietary interventions. Neurochem Int.

[27] Guan X.M., Yu H., Trumbauer M., Frazier E., Van der Ploeg L.H., Chen H. (1998). Induction of neuropeptide Y expression in dorsomedial hypothalamus of diet-induced obese mice. Neuroreport.

[28] Bergen H.T., Mizuno T., Taylor J., Mobbs C.V. (1999). Resistance to diet-induced obesity is associated with increased proopiomelanocortin mRNA and decreased neuropeptide Y mRNA in the hypothalamus. Brain Res.

[29] Ziotopoulou M., Mantzoros C.S., Hileman S.M., Flier J.S. (2000). Differential expression of hypothalamic neuropeptides in the early phase of diet-induced obesity in mice. Am J Physiol Endocrinol Metab.

[30] Levin B.E., Dunn-Meynell A.A. (1997). Dysregulation of arcuate nucleus preproneuropeptide Y mRNA in diet-induced obese rats. Am J Physiol Regul Integr Comp Physiol.

[bib31] Takahashi N., Patel H.R., Qi Y., Dushay J., Ahima R.S. (2002). Divergent effects of leptin in mice susceptible or resistant to obesity. Horm Metab Res.

[32] Huang X.F., Han M., Storlien L.H. (2003). The level of NPY receptor mRNA expression in diet-induced obese and resistant mice. Brain Res Mol Brain Res.

[33] Huang X.F., Xin X., McLennan P., Storlien L. (2004). Role of fat amount and type in ameliorating diet-induced obesity: insights at the level of hypothalamic arcuate nucleus leptin receptor, neuropeptide Y and pro-opiomelanocortin mRNA expression. Diabetes Obes Metab.

[34] Park E.S., Yi S.J., Kim J.S., Lee H.S., Lee I.S., Seong J.K. (2004). Changes in orexin-A and neuropeptide Y expression in the hypothalamus of the fasted and high-fat diet fed rats. J Vet Sci.

[35] Beck B. (2006). Neuropeptide Y in normal eating and in genetic and dietary-induced obesity. Phil Trans R Soc Lond B Biol Sci.

[36] Wang C., Yang N., Wu S., Liu L., Sun X., Nie S. (2007). Difference of NPY and its receptor gene expressions between obesity and obesity-resistant rats in response to high-fat diet. Horm Metab Res.

[37] Wang H., Storlien L.H., Huang X.F. (2002). Effects of dietary fat types on body fatness, leptin, and ARC leptin receptor, NPY, and AgRP mRNA expression. Am J Physiol Endocrinol Metab.

[38] Patel H.R., Qi Y., Hawkins E.J., Hileman S.M., Elmquist J.K., Imai Y. (2006). Neuropeptide Y deficiency attenuates responses to fasting and high-fat diet in obesity-prone mice. Diabetes.

[39] Bi S., Chen J., Behles R.R., Hyun J., Kopin A.S., Moran T.H. (2007). Differential body weight and feeding responses to high-fat diets in rats and mice lacking cholecystokinin 1 receptors. Am J Physiol Regul Integr Comp Physiol.

[40] Staszkiewicz J., Horswell R., Argyropoulos G. (2007). Chronic consumption of a low-fat diet leads to increased hypothalamic agouti-related protein and reduced leptin. Nutrition.

[41] Kaushik P., Gorin F., Vali S. (2007). Dynamics of tyrosine hydroxylase mediated regulation of dopamine synthesis. J Comput Neurosci.

[42] Zheng G., Dwoskin L.P., Crooks P.A. (2006). Vesicular monoamine transporter 2: role as a novel target for drug development. AAPS J.

[43] Perez R.G., Waymire J.C., Lin E., Liu J.J., Guo F., Zigmond M.J. (2002). A role for alpha-synuclein in the regulation of dopamine biosynthesis. J Neurosci.

[44] Senior S.L., Ninkina N., Deacon R., Bannerman D., Buchman V.L., Cragg S.J. (2008). Increased striatal dopamine release and hyperdopaminergic-like behaviour in mice lacking both alpha-synuclein and gamma-synuclein. Eur J Neurosci.

[45] Meister B., Elde R. (1993). Dopamine transporter mRNA in neurons of the rat hypothalamus. Neuroendocrinology.

[46] Garrett M.C., Soares-da-Silva P. (1990). Role of type A and B monoamine oxidase on the formation of 3,4-dihydroxyphenylacetic acid (DOPAC) in tissues from the brain of the rat. Neuropharmacology.

[47] Missale C., Nash S.R., Robinson S.W., Jaber M., Caron M.G. (1998). Dopamine receptors: from structure to function. Physiol Rev.

[48] Baptista T., Araujo de Baptista E., Ying Kin N.M., Beaulieu S., Walker D., Joober R. (2002). Comparative effects of the antipsychotics sulpiride or risperidone in rats. I: bodyweight, food intake, body composition, hormones and glucose tolerance. Brain Res.

[49] Kuo D.Y. (2002). Co-administration of dopamine D1 and D2 agonists additively decreases daily food intake, body weight and hypothalamic neuropeptide Y level in rats. J Biomed Sci.

[50] Yang Z.J., Meguid M.M. (1995). LHA dopaminergic activity in obese and lean Zucker rats. Neuroreport.

[51] Fetissov S.O., Meguid M.M., Sato T., Zhang L.H. (2002). Expression of dopaminergic receptors in the hypothalamus of lean and obese Zucker rats and food intake. Am J Physiol Regul Integr Comp Physiol.

[52] Nicola S.M. (2000). Dopaminergic modulation of neuronal excitability in the striatum and nucleus accumbens. Annu Rev Neurosci.

[53] Martel P., Fantino M. (1996). Influence of the amount of food ingested on mesolimbic dopaminergic system activity: a microdialysis study. Pharmacol Biochem Behav.

[54] Comings D.E., Blum K. (2000). Reward deficiency syndrome: genetic aspects of behavioral disorders. Prog Brain Res.

[55] Geiger B.M., Behr G.G., Frank L.E., Caldera-Siu A.D., Beinfeld M.C., Kokkotou E.G. (2008). Evidence for defective mesolimbic dopamine exocytosis in obesity-prone rats. FASEB J.

[56] Chan M.-Y., Zhao Y., Heng C.-K. (2008). Sequential responses to high-fat and high-calorie feeding in an obese mouse model. Obesity.

[57] Krugel U., Schraft T., Kittner H., Kiess W., Illes P. (2003). Basal and feeding-evoked dopamine release in the rat nucleus accumbens is depressed by leptin. Eur J Pharmacol.

[58] Fulton S., Woodside B., Shizgal P. (2000). Modulation of brain reward circuitry by leptin. Science.

[59] Figlewicz D.P., Bennett J., Evans S.B., Kaiyala K., Sipols A.J., Benoit S.C. (2004). Intraventricular insulin and leptin reverse place preference conditioned with high-fat diet in rats. Behav Neurosci.

[60] Blouquit M.F., Gripois D., Roffi J. (1996). Influence of cold exposure on dopamine content in rat brown adipose tissue. Horm Metab Res.

[61] Ootsuka Y., Heidbreder C.A., Hagan J.J., Blessing W.W. (2007). Dopamine D2 receptor stimulation inhibits cold-initiated thermogenesis in brown adipose tissue in conscious rats. Neuroscience.

